# A review of skin disease in military soldiers: challenges and potential solutions

**DOI:** 10.1080/07853890.2023.2267425

**Published:** 2023-10-16

**Authors:** Amit Singal, Shari R. Lipner

**Affiliations:** a Rutgers NJ Medical School, Newark, NJ, USA; b Department of Dermatology, Weill Cornell Medicine, New York, NY, USA

**Keywords:** Military medicine, military personnel, skin diseases, dermatological conditions, operational dermatology

## Abstract

**Introduction:** Military soldiers comprised 1,195 million United States active-duty members and 778,000 reserve members in 9/2021. Soldiers are often exposed to drastic climates, environments, and living conditions which may make them more susceptible to cutaneous diseases.

Methods: A PubMed search of studies published between 1/1/2002 – 8/30/2022, using MeSH terms: ((("Military Personnel"[Majr]) OR "Military Hygiene"[Majr])) OR "Military Medicine"[Majr]) AND "Skin Diseases"[Majr]), the reference lists of select articles, and other applicable sources were reviewed to identify articles on skin conditions affecting military soldiers and treatment options.

**Discussion:** In this article, we review skin conditions that affect military soldiers in both the deployed and non-deployed settings including infectious diseases, arthropod associated diseases, sexually transmitted infections, ultraviolet radiation related skin disease, acne, diseases of hair and hair follicles, dermatitis, onychocryptosis, and conditions caused by extreme weather conditions and occupational exposures. We also discuss treatment options and prevention methods as they relate to military settings.

**Conclusion:** Dermatological conditions can considerably impact soldiers’ wellbeing and military performance, often lead to evacuation of military personnel, and are associated with high financial costs. Cutaneous disease is one of most common reasons for soldiers to seek medical care and may cause significant morbidity. Serving in the military often impacts and limits treatment options.

## Key messages

1. Military soldiers are a unique population exposed to crowded living conditions, reduced opportunity for personal hygiene, environmental contamination, strict grooming practices, and skin injury which may contribute to infectious and arthropod associated skin, hair, and nail diseases in soldiers.
2. Treatment options for skin diseases that are commonly used in the general population may have implications for military duties that should be considered before treatment initiation in soldiers.

## Introduction

Military soldiers are a unique population exposed to crowded living conditions, environmental contamination, skin injury, and temperature extremes, which can make them more susceptible to dermatologic disease [1–3]. The military represents a substantial population, with 1195 million United States (US) active-duty service members and 778,000 reserve members in 9/2021 [4]. Skin diseases often lead to evacuation of military personnel from combat theater and affect soldier training and morale [5,6]. Cutaneous disease related disruptions in the military not only hinder military operations but are also associated with high financial costs. In a study on teledermatology consults of deployed US soldiers in Iraq 1/2005–1/2009, the cost of evacuating soldiers back to the US was estimated at $562,380 and the cost of in-person dermatology evaluations in Iraq was $416,000 [7]. Challenges of treating skin conditions in deployed soldiers include slow turnaround time for pathology specimens, lack of access to dermatological care, and arranging follow up care [8].

While mortality from skin disease in soldiers is low, dermatologic conditions in the military setting comprise a large proportion of soldier morbidity. In a review of skin conditions in military peacekeeping operations, military physician visits for skin conditions ranged from 9.3% (during the Gulf war) to 25.2% (in an East Timor deployment) [9]. In US soldiers specifically, 10% (429,837 cases) of deployed setting medical diagnoses 2008–2015, were dermatological [10].

The climate, environment, and living conditions in which the soldiers serve may affect their spectrum of skin diseases (Tables 1 and 2) [5,6,9,11,12]. For example, outpatient visits for cutaneous disease during World War II was 15–25% in temperate climates, and 75% in tropical climates [13–15]. Given the large percentage of dermatology consults in deployed soldiers, impact on soldiers’ wellbeing and ability to perform, and associated costs, US military dermatologists have recommended that dermatologists’ deployment as theater consultants would help reduce costs and lost workdays due to cutaneous disease [13]. Prior reviews of dermatoses affecting military populations have focused solely on military operations [9] or deployed settings in a specific military population [16]. However, a review discussing dermatological conditions in both deployed and base settings inclusive of worldwide militaries is lacking. In this review, we highlight common skin disorders that affect military soldiers of different militaries in both deployed and base settings, challenges they present to military personnel and military operations, and potential solutions.

**Table 1.** Prevalence of skin disease in different military deployments.

Army	Name of Operation	Deployment location	Climate	Type of mission	Length of time	Number of dermatology diagnoses	Dermatological diagnoses % of total medical diagnoses	Bacterial infection (% of skin diagnoses)	Fungal Infection (% of skin diagnoses)	Viral infection (% of skin diagnoses)	Dermatitis and eczematous conditions (% of skin diagnoses)	Acne (% of skin diagnoses)	Diseases of hair and hair follicles (% of skin diagnoses)	Skin Cancer	Insect Bites (% of skin diagnoses)
US [19] (Army field Hospital in Saudi Arabia)	Persian Gulf War	Saudi Arabia	Hot and Dry	Warfare	2/1991–3/1991	81	N/A	7.2%	13%	11.5%^a^ (includes Viral infection, other and Wart)	16.5% (includes: AD, CD, eczema, seborrheic dermatitis)	4.3%	4.3%	0%	N/A
US [21]	Vietnam War	Vietnam	Hot and Humid	Warfare	1965–1970	4166	12.2%	21.2%^a^	3%^a^	N/A	8.5%^a^	N/A	N/A	N/A	N/A
Australian [5]	N/A	East Timor	Hot and humid	Peacekeeping	93 d (1999)	193^b^	25.2%	15.5%	23.3%	17%	16%	1%	0.5%	N/A	N/A
US [7]	OIF	Iraq	Hot and Dry	N/A	1/2005–1/2009	2197	N/A	7%	7%	N/A	13%	N/A	N/A	N/A	N/A
US [28]	OIF	Iraq	Hot and Dry	N/A	1/15/2008–7/15/2008	2696	N/A	6%	6%	5%^a^ (Includes viral disease and warts)	18%^a^ (includes eczema and seborrheic dermatitis)	9%	3%^a^ (includes alopecia and pseudo folliculitis barbae)	8%	1%^a^
United Nations (different countries) [18]	United Nations and African Mission in Darfur (UNAMID)	Darfur, Sudan	Hot and dry climate	peacekeeping	3/2014–2/2015	542	N/A	N/A	22.5%	N/A	38.7%	10.7%	N/A	N/A	N/A
United Nations [17]	United Nations Interim Forces in Lebanon (UNIFIL)	Lebanon	Hot and dry	peacekeeping	1/2018–5/2019	549 (experienced by 399 military personnel and 150 civilians)	N/A	9.3%	13.3%	7.5%	27.1%	6.6%	5.1%	N/A	2.2%
US [10]	Many	Afghanistan, Iraq, other regions of Middle East, Africa, Asia, Americas (all deployments during timeframe)	Multiple	Multiple	2008–2015	429,837	10%	7.5%	11.6%	N/A	8.5%^a^ (Contact Dermatitis = 7.7% Eczematous dermatitis 0.8%)	5.5%	8.1%	0.02%	N/A
Britain [20]	HERRICK	Afghanistan	Hot and dry	N/A	7/2010	365	20%	12%	25%	8%	N/A	N/A	N/A	N/A	N/A

AD: Atopic dermatitis (AD); CS: Contact dermatitis; OIF: Operation Iraqi Freedom (OIF).

^a^ Percentages were calculated from information provided in publication for comparison with other publications.

^b^Skin diagnoses of different conditions were added up to find total number of skin disease diagnoses.

Table 1 shows the prevalence of different skin diseases that affected military soldiers in different deployments. Articles used in this table were first identified conducting a PubMed search of Major MeSH terms “Military Personnel” OR “Military Hygiene” OR “Military Medicine” AND “Skin Diseases” From 1/1/2002–8/30/2022. Articles that included prevalence of skin disease in military soldiers in deployed settings were added to this table. In addition, references of relevant articles were queried to expand search and some, but not all, of relevant articles were included. [5,7,10,17–21,28].

**Table 2.** Prevalence of skin disease in non-deployed settings.

Army	Climate	Length of time	Number of dermatology diagnoses	Dermatological diagnoses % of total medical diagnoses	Fungal Infection (% of skin diagnoses)	Bacterial infection (% of skin diagnoses)	Dermatitis and eczema (% of skin diagnoses)	Viral infection (% of skin diagnoses)	Acne (% of skin diagnoses)	Diseases of hair and hair follicles (% of skin diagnoses)	Skin Cancer (% of skin diagnoses)	Insect bite reactions
Norwegian [11]		8 months 9/1996–5/1997	222	16%	5%	N/A	18.9%	Total: 9%^a^ Including (Common warts and HSV)	10.4%	N/A	N/A	N/A
Turkish [22]	Hot and humid (during time of study)	2 d	609^c^ (experienced by 183 military personnel)	N/A	15.9%^a^	10.6%^a^	21.8%^a^ (includes Dyshidrotic eczema, Intertrigo, Pityriasis capitis simplex, CD, and Asteatotic eczema)	1%^a^	5.6%^a^	3.8%^a^	12.6%^a^	N/A
Korean [23]		4/–9/2010	1081^b^ (experienced by 798 military personnel)	N/A	24.5%^a^ Includes:Tinea pedis, onychomycosis, tinea cruris, P. versicolor, tinea corporis, other)	3.7%^a^ (Includes: Bacterial folliculities, and Pitted keratolysis)	13.4%^a^ (Includes: Atopic, Seborrheic, Contact, Hand, and Other dermatitis)	5.7%^a^	43.7%^a^	3.2%^a^	N/A	0.4%^a^
Singaporean [12]		2 years (2007–2009)	9176	N/A	28%	6%)	24%^a^ (Includes: Nonspecific dermatitis, CD, and Endogenous eczema)	1%	5%	3.2%^a^ (Includes: folliculitis and hair disorder)	N/A	7%

CD: Contact dermatitis (CD); HSV: Herpes simplex virus (HSV).

Table 2 shows the prevalence of different skin diseases affecting military soldiers in studies from non-deployed settings. Articles used in this table were first identified conducting a PubMed search of Major MeSH terms “Military Personnel” OR “Military Hygiene” OR “Military Medicine” AND “Skin Diseases” From 1/1/2002–8/30/2022. Articles that included prevalence of skin disease in military soldiers in non-deployed settings were added to this table. In addition, references of relevant articles were queried to expand search and some, but not all, of relevant articles were included [11,12,22,23].

^a^ Percentages were calculated from information provided in publication for comparison with other publications.

^b^ Skin diagnoses of different conditions were added up to find total number of skin disease diagnoses.

c Skin diagnoses of different conditions in this cited study were added to find total number of skin disease diagnoses.

## Methods

A PubMed search was used to identify studies published between 1/1/2002 and 8/30/2022 in English, using MeSH terms search: (((‘Military Personnel’[Majr]) OR ‘Military Hygiene’ [Majr])) OR ‘Military Medicine’ [Majr]) AND ‘Skin Diseases’[Majr]). 312 PubMed results were identified. Article type selection included the following subcategories: Systematic reviews, clinical study, clinical trial, comparative studies, multicenter studies, observational studies, randomized controlled trials, and review articles. Articles evaluating specific treatments, focused non-soldier populations or non-dermatology conditions were excluded. Reviews covering topics sufficiently covered by studies were excluded. The reference list of select publications was used to expand the search. Relevant articles found outside of the primary search methods were evaluated and included and cited in the reference list to supplement discussion. Articles on monkeypox and the Coronavirus disease 2019 (COVID-19) were searched for outside of the primary search method, as to highlight current public health issues ongoing at the time of manuscript drafting.

## Discussion

The studies included in this review highlight differences in prevalence of dermatoses in military populations by environmental settings. In studies of deployed soldiers serving in hot and dry climates, dermatitis and eczematous conditions were most common accounting for 13%–38.7% of all skin dermatoses [7,17–20]. Studies of deployed soldiers serving in hot and humid conditions showed the highest prevalence of bacterial (21.2%) and fungal (23.3%) infections being the most common dermatologic conditions (Table 1) [5,21]. In the non-deployed setting, dermatitis and eczematous conditions accounted for 13.4%–24%, fungal infections accounted for 5%–28%, and acne accounted for 5%–43.7% of all skin conditions (Table 2) [11,12,22,23].

## Infectious diseases

### Bacterial skin infections

Bacterial skin infections including those caused by Methicillin-Resistant Staphylococcus aureus (MRSA) are common during military training [24]. Crowded living conditions, reduced opportunity for personal hygiene, environmental contamination, and skin injury contribute to higher rates of skin and soft tissue infections (SSTIs) in military recruits compared to all military personnel [1–3]. In a study of SSTI’s in 772 military trainees serving in a joint base in San Antonino-Lackland, Texas, 10/1/2012–12/31/2014, there were 254 culture positive SSTI cases, of which 43.3% grew MRSA, 26.8% Methicillin-sensitive S. aureus, and 7.2% and 2% grew other gram-negative and gram-positive cocci respectively (16.6% grew usual skin flora and 6.3% had no growth) [24]. A 2012–2014 whole genome sequencing study of US Army Infantry trainees with confirmed MRSA SSTI’s, showed intra- and inter-class transmission of MRSA among military soldiers, suggesting the introduction of multiple strains of MRSA, as well as person to person transmission in military settings [25].

**Figure 1.**
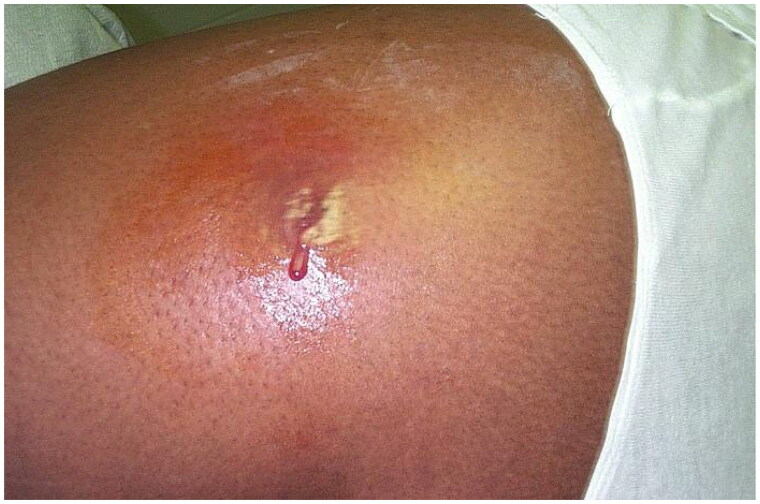
Cutaneous abscess on the lateral thigh. There is erythema, fluctuance, and purulence. Photo by Bruno Coignard, M.D.; Jeff Hageman, M.H.S. Reprinted from public domain Centers for disease Control and Prevention public Health Image Library; Retrieved from https://phil.cdc.gov/details.aspx?pid=7826.

Two surveillance studies of active US military personnel reported over 490 thousand SSTI cases 2013–2020. However, between 2013–2016 and 2016–2020 crude incidence rates decreased from 558.2/10,000 person years (p-yrs) to 352.8/10,000 p-yrs respectively with a shift from ‘other’ SSTI’s (including cellulitis, impetigo, and pyoderma) to cellulitis/abscess as the most common SSTI (Figure 1). Carbuncles, furuncles and ‘other’ SSTI’s occurred more frequently in Black service members. In the 2013–2016 cohort, incidence rates of carbuncles/furuncles and ‘other’ SSTI’s in Black service members were 37.4 per 10,000 p-yrs and 911.2 per 10,000 p-yrs, respectively, vs. 26 per 10,000 p-yrs and 276 per 10,000 p-yrs, respectively, in the total study population. In the 2016–2020 cohort, incidence rates of carbuncles/furuncles and ‘other’ SSTI’s in Black service members were 27.5 per 10,000 p-yrs and 120.6 per 10,000 p-yrs respectively vs. 18.7 per 10,000 p-yrs and 105.9 per 10,000 p-yrs, respectively, in the total study population. Cellulitis/abscess were more common in White service members. In the 2013–2016 cohort, incidence rates of cellulitis/abscess in White service members were 273.4 per 10,000 p-yrs vs. 256 per 10,000 p-yrs in the total study population. In the 2016–2020 cohort, incidence rates of cellulitis/abscess in White service members were 243.7 per 10,000 p-yrs vs. 227.7 per 10,000 p-yrs in the total study population [3,26]. Incidence of SSTI’s was higher in female vs. male military personnel 2016–2020 (Female SSTI rate of 397.9 vs. 343.9 in males) [26]. There was also a higher proportion of MRSA SSTI cases in female vs. male military trainees serving in a joint base in Texas 2012–2014, (81% in females vs. 58% in males) [24]. A questionnaire-based study of US military personnel mid-deployment 8/2007 − 7/2008, reported that in any branch besides the marines, females had an increased risk of reporting SSTI’s (*p* = .01), with the army having the largest sample size (95.3% of total study participants) and 6.9% of female army personnel reporting SSTI’s compared to 4.7% for males [27].

Incidence rates of bacterial skin infections during Operation Iraqi Freedom (OIF), and East Timor Deployment, and the Vietnam war were 6%, 15%, and 21.2% of all skin disease diagnoses, respectively (Table 1) [5,21,28]. East Timor’s and Vietnam’s hot and humid climates, likely provide a favorable environment for the development of bacterial infections [5,21]. In Iraq, bacterial infections presented in all patients as painful abscess on the extremities or back [28]. In East Timor, bacterial infections included superficial folliculitis and pitted keratolysis. Superficial folliculitis often progressed to furuncles, and less often to carbuncles [5].

In a review of SSTIs in the military published in 2013, Lamb et al. recommended hygiene practices, education, and isolation of cases, as strategies to prevent the spread of bacterial infection in the military. Early recognition and treatment were highlighted as key for treating bacterial SSTI’s in military soldiers. For superficial uncomplicated infections, topical agents are generally recommended. However, in deployed settings, systemic antibiotics are recommended due to the barriers of applying topical agents in this environment. If necrotizing fasciitis occurs in soldiers on operations, recommended treatments are linezolid, meropenem, and clindamycin for broad coverage. Military soldiers may be exposed to less common pathogens compared to the general population due to their changing environments. For example, *Aeromonas hydrophilia* or *vibrio* species may be responsible for cellulitis from marine-related injuries. Testing for Panton-Valentine Leukocidin *S. Aureus* and other resistant or unusual pathogens may be considered in soldiers deployed to foreign countries. Methicillin resistance should be considered when treating both uncomplicated and complicated infections [29].

### Fungal infections

Dermatophytosis was the most common dermatologic diagnosis in a retrospective study of 429,837 dermatologic diagnoses in deployed US soldiers 2008–2015, accounting for 11.6% of all dermatologic diagnoses, which is higher than the dermatophytosis rate in US civilian non-dermatologist and dermatologist diagnoses (3.9% and 1.8% of the 431,870,000 and 296,100,000 total dermatologic diagnoses, respectively) [10]. Hot and humid environments in some military deployments likely contribute to a higher prevalence of dermatophyte infections (Table 1). Fungal skin infections were the most common skin condition in two military cohorts serving in East Timor 1999–2000 and in Singapore 2007–2009, which share similar tropical environments [5,12]. In the East Timor deployment, 45 diagnoses of dermatophyte infections included tinea pedis (38/45), tinea corporis and tinea cruris (6/45), and pityrosporum folliculitis (1/45), with *Trichophytum rubrum* isolated from 90% culture positive cases [5]. Fungal infections are also a significant cause of morbidity in non-tropical environments. In a Chinese Peacekeeping mission in Lebanon, which has Mediterranean climate conditions, cutaneous mycosis was the second most common diagnosis accounting for 13.3% of the 549 dermatologic diagnoses, with tinea corporis and tinea pedis being the most common types (5.3% and 4.7% of total dermatological diagnoses, respectively) [17].

**Figure 2.**
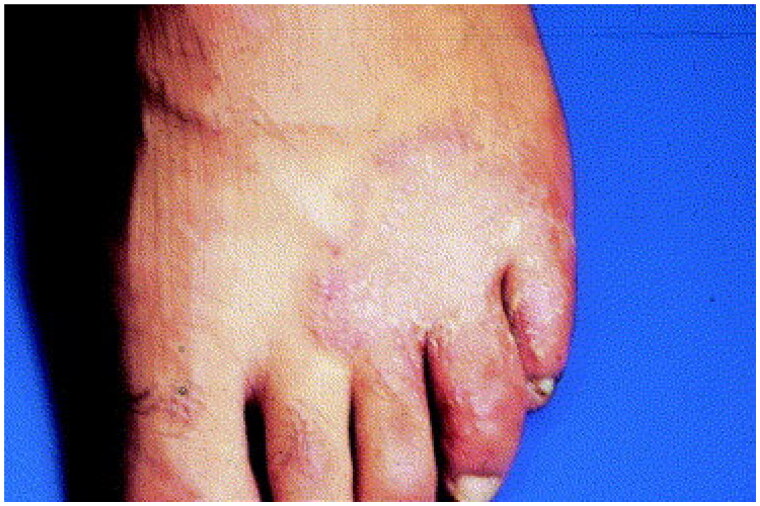
Tinea pedis. Annular scaly plaques involving the dorsal foot and web spaces. Reprinted with permission from: Oumeish and Parish [40], with permission from Elsevier.

Tinea pedis may lead to toenail onychomycosis, and secondary infections (Figure 2) [30–32]. The worldwide prevalence of tinea pedis is approximately 15% [33]. In a 17-year surveillance study of the US military 2000–2016, the overall incidence rate of tinea pedis was 84.0 cases per 10,000 p-yrs. The overall incidence rate was 17.4% higher in male vs. female service members (85.9 cases per 10,000 p-yrs vs. 73.1 cases per 10,000 p-yrs, respectively), and Black and Hispanic service members had the highest overall incidence rates (109.9 cases per 10,000 p-yrs and 91.3 cases per 10,000 p-yrs, respectively) [31]. A cross-sectional study of tinea pedis in 223 Israeli soldiers conducted during a 2-month hot and dry summer period that was published in 2005, showed a 60.1% (*n* = 134/223) clinical prevalence (positive clinical skin examination) and 27.3% (*n* = 61/223) mycological prevalence (positive cultures) of tinea pedis. Male gender (*p* = .002), setting of military training (*p* < .001), and infrequent sock changes (*p* = .043) were associated with a higher prevalence of tinea pedis on univariate analysis [34]. Another cross-sectional study of tinea pedis in 729 male soldiers and 279 male civilians employed by the Georgian Defense Forces, published in 2021, reported a clinical and mycological prevalence of tinea pedis of 46.4% (*n* = 340/729) and 24.25% (*n* = 184/729), respectively, in soldiers and 21.86% and 13.98%, respectively, in civilians. In total, 10.13% (*n* = 74/729) of soldiers had toenail onychomycosis. History of tinea pedis prior to military service (*p* = .159), wearing military boots for 14 h every day (*p* = .105), and using communal showers (*p* = .074) were associated with a higher prevalence of tinea pedis. Wearing two or more pairs of military boots, which lessens moisture and humidity, was associated with a decreased risk of tinea pedis (*p* = −0.354) [35]. A Japanese study published in 1995 analyzed the relationship between the interdigital space and tinea pedis in 74 soldiers undergoing specialized training in Texas, USA. Soldiers were divided into 3 groups based on the width of their interdigital spaces (I = wide, II = fairly wide, III = closed). The prevalence of tinea pedis in groups I, II and III was 30% (*n* = 3) vs. 77% (*n* = 19) vs. 90% (*n* = 27), respectively (*p* < .01), with severity medians of 2.3, 4.3, and 4.2, respectively (Spearman *r* = 0.12, *p* < .32) [36], suggesting that smaller interdigital space width conferred greater risk and severity of tinea pedis.

Preventative efforts, such as education on foot and skin health maintenance practices, and administration of topical antifungals may help decrease the incidence of tinea pedis [18,31,36–39]. In a study of 169 Australian troops serving in East Timor 9/27/1999-2/20/2000, all troops were issued preventative foot powder (83% talc, 10% starch and 3% salicylic acid) and topical treatments were used for cases of tinea corporis and tinea cruris [5]. In a review of SSTIs in the military published in 2013, Lamb et al. recommended treatment with topical antifungals (such as ketoconazole), terbinafine creams, or oral terbinafine for more resilient tinea pedis infections [29].

### Severe acute respiratory syndrome coronavirus-2 (SARS-CoV-2)

In 2019, an outbreak of SARS-CoV-2, responsible for the COVID-19, resulted in a worldwide pandemic concerning to both civilian and military populations [41]. COVID-19 is a multi-organ disease than can also affect the skin [42]. The six main clinical patterns to the COVID-19 associated cutaneous manifestations include urticarial rash, maculopapular rash, papulovesicular exanthem, chilblain-like lesions, livedo reticularis/racemosa-like pattern, and vasculitic pattern [43]. In 2021, 0.4% of the 131,694 medical encounters in US service members deployed to Southwest Asia, the Middle East, and Africa were for COVID-19, with skin diseases accounting for 5.9% of servicemen’s and 3.9% of servicewomen’s medical encounters [44]. In an Italian study on the cutaneous manifestations of COVID-19 in 81 military patients and 15 civilians hospitalized in COVID-19 non-intensive care wards 3/16/2020–5/4/2020, 25 (31%) military patients and 9 civilians (60%) developed skin manifestations. The most common dermatologic diagnosis was xerosis in 11 (13.6%) military patients and 4 (26.7%) civilians. Other diagnoses included seborrheic dermatitis (3 cases in military group and 1 in civilian group), irritant CD (ICD) (3 cases in military group and 2 in civilian group), morbilliform rash (3 cases in military group and 1 in civilian group), petechial rash (2 cases in military group and 1 in civilian group), and urticaria (3 cases in military group and 0 in civilian group) [42].

COVID-19 vaccines are effective in preventing systemic symptoms and cutaneous reactions, [45,46] but there was some resistance to vaccination within the military prior to mandates. A survey-based study 11/2020–1/2021 of 816 individuals associated with Wright-Patterson Air Force (AF) Base in the US (not limited to soldiers), reported that younger respondents (defined as 30 years or younger) were more likely to be vaccine hesitant than older (older than 30 years old) respondents (38% vs. 18% *p* < .001) [47]. A case control study of 441,379 unvaccinated and fully vaccinated US military personnel tested for SARS-CoV-2 1/1/2021–9/24/2021, investigated two time periods before and after the new delta variant of SARS-CoV-2 emerged, the pre-delta period (1/1/2021–5/31/2021), and the delta period (6/19/2021–9/24/2021) respectively. Noteworthy, mandatory COVID-19 vaccination requirements for all military members went into effect on 8/24/2021. In the pre-delta period, 1% (*n* = 466/38,968) of positive SARS-CoV-2 tests and 14% (*n* = 34,243/251,288) of negative SARS-CoV-2 tests were reported in vaccinated military personnel and 99% (*n* = 38,501/38,968) of positive SARS-CoV-2 tests and 86% (*n* = 217,032/251,288) of negative SARS-CoV-2 tests were reported in unvaccinated military personnel. In the delta period, 38% (*n* = 10,041/26,087) of positive SARS-CoV-2 tests and 65% (*n* = 81,486/125,036) of negative SARS-CoV-2 tests were reported in vaccinated military personnel and 62% (*n* = 16,044/26,087) of positive SARS-CoV-2 tests and 35% (*n* = 43,537/125,036) of negative SARS-CoV-2 tests were reported in unvaccinated military personnel [46].

### Parasitic infections

Parasitic skin diseases may affect military soldiers who are often exposed to non-native environments and close living conditions. Parasitic infections accounted for 1% of the 2696 skin diagnoses in a military dermatology clinic in Iraq 1/15/2008–7/15/2008, and for 2.8% of the 81 skin diagnoses made in a military evacuation hospital in Saudi Arabia 2/3/1991–3/8/1991 [19,28]. In Iraq (1/15/2008-7/15/2008), these included scabies (*n* = 19), cutaneous larvae (*n* = 3), and cutaneous leishmaniasis (*n* = 1) [28]. Military prevention of insect bites has commonly includes insecticide-treated clothing (such as permethrin, and DEET), and bed nets which are evidenced based [48,49].

### Cutaneous leishmaniasis (CL)

**Figure 3.**
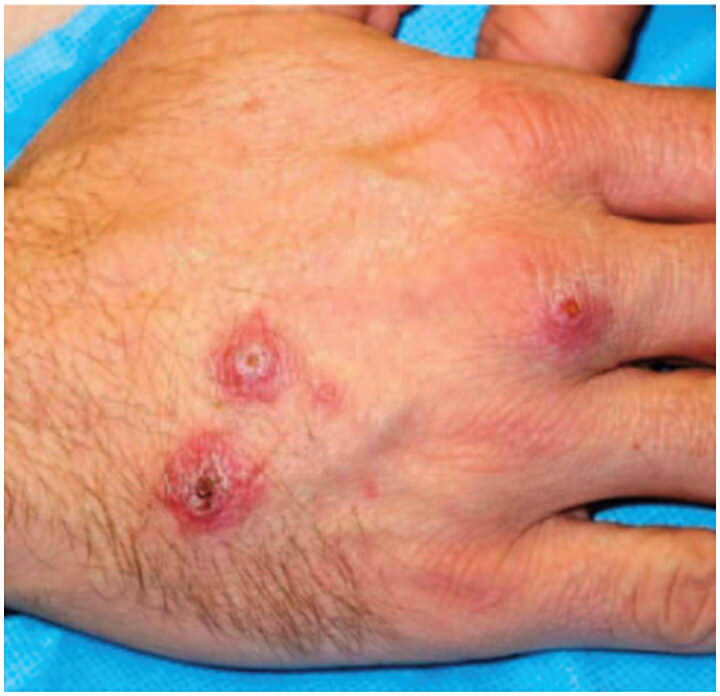
Cutaneous leishmaniasis. Central domed, eroded, and crusted erythematous nodules on the right dorsal hand of a soldier returning from Iraq deployment. This case has been previously reported. Reprinted with permission from Pehoushek et al. [51], with permission from Elsevier.

**Figure 4.**
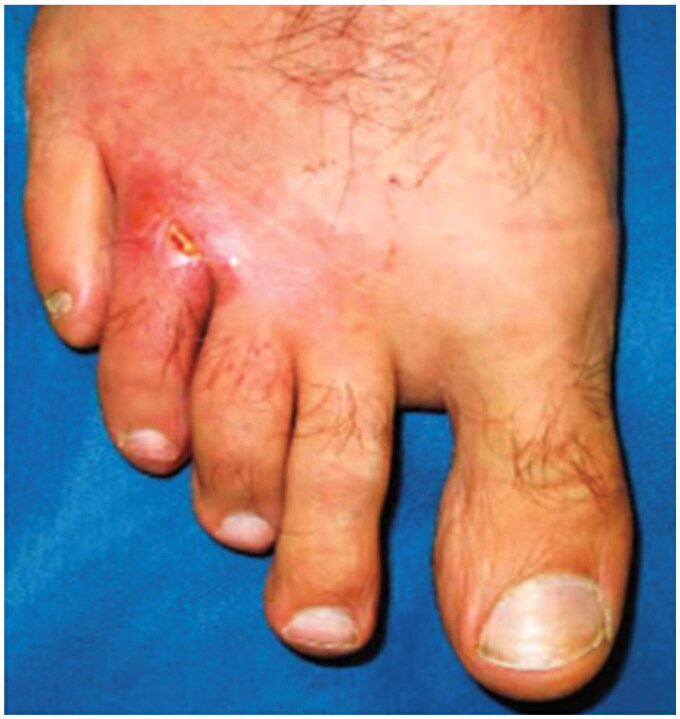
Cutaneous leishmaniasis. Ulcerated plaque on the right fourth toe and third web space. This case has been previously reported. Reprinted with permission from: Pehoushek et al. [51], with permission from Elsevier.

Leishmaniasis is a sandfly borne parasitic infection that is transmissible between humans, which may affect the skin, mucosal membranes, and the reticuloendothelial system. The presentation can vary from asymptomatic, to self-limited CL, to visceral disease which is life threatening [50]. The infection may not be immediately apparent and is often diagnosed after the soldier has returned home from deployment. In addition, CL presents similarly to other skin conditions making diagnosis challenging (Figures 3 and 4) [51].

Leishmaniasis is endemic to many areas globally. On 1/8/2022, the World Health Organization (WHO) reported that 95% of CL cases occur in the Americas, Mediterranean basin, and Middle East and Central Asia [52]. CL outbreaks were reported in military soldiers, often after returning from missions in deployed settings [53–56]. Over 500 CL cases were diagnosed in military personnel returning from deployment in Afghanistan, Iraq and Kuwait 2002–2004 [57]. A 2004 briefing of military operations in Iraq estimated that 10% of military persons deployed to the Persian Gulf area might have been infected with leishmaniasis [8,51].

Vector control program, and personal protective equipment, including long-sleeved uniforms treated with permethrin and mosquito repellents, may be used for the prevention of leishmaniasis in endemic areas [58]. Yet, in a cross-sectional study of the use of personal protection in 300 Columbian soldiers who trained 10/2014–12/2014 in leishmaniasis-endemic areas, only 23% of soldiers reported complete use of personal protection measures (defined as complete military uniform treated with permethrin, rolled down long sleeves, cap and boots wearing, and application of mosquito repellant in all exposed body areas) [59]. The only Food and Drug Administration (FDA) approved leishmaniasis medication in the US currently are Liposomal amphotericin B (IV infusion) for visceral leishmaniasis (VL), since 1997, and oral miltefosine (2, 50 mg oral capsules/day for 28 d) for CL, VL, and mucosal leishmaniasis (ML), since 2014 [60]. Despite not being FDA approved for use in the US, sodium stibogluconate (SSG), with a standard daily dose of 20 mg/Kg (IV or IM) for 20 d for CL and 28 d for ML and VL, is a traditional treatment for simple CL lesions used since the 1940s in many parts of the world [60]. A 2002 randomized double-blind study of SSG treatment for CL in 38 US military personnel infected with CL while abroad 8/1990–3/1991 and 9/1996–6/2001, there was no difference (*p* = .79) between treatment with SSG for 10 d vs. 20 d at 20 mg/kg, with cure rates of 100% (19/19) and 95% (*n* = 18/19), respectively, suggesting that 10 d of 20 mg/kg SSG may be sufficient treatment for CL [61]. Some treatments are only effective against certain leishmaniasis infections and species, and individualized treatment regimens should include species identification [60]. There is limited data supporting treatment regimens for leishmaniasis, meriting further research.

### Scabies

**Figure 5.**
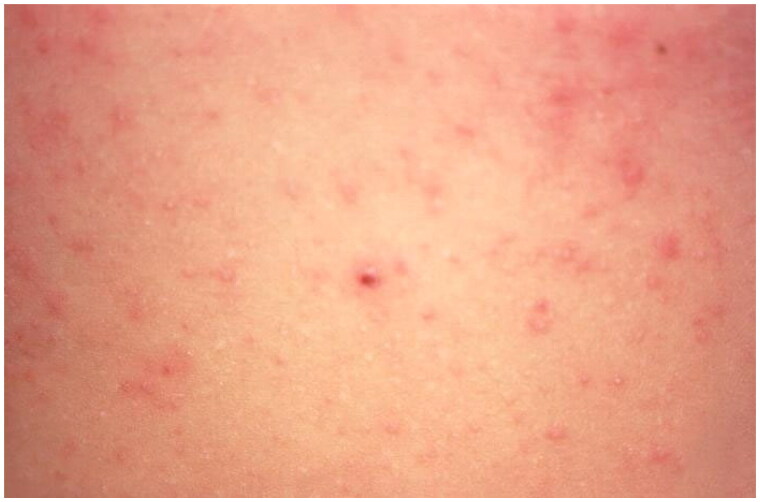
Scabies. Erythematous papules on the abdomen due to hypersensitivity to the scabies mite. Image by Joe Miller. Reprinted from public domain Centers for disease Control and Prevention public Health Image Library; Retrieved from https://phil.cdc.gov/details.aspx?pid=15382.

Scabies is a highly contagious parasitic skin disease, which can cause significant morbidity due to severe itch (Figure 5) [62]. Scabies was very common in the military setting in the eighteenth century and during a 1917 outbreak in the British military. However, incidence has since decreased, likely due to improved military hygiene, preventative health measures, and effective treatments [63]. In a dermatology clinic in Iraq 1/15/2008–7/15/2008, there were only 19 cases of scabies from 2696 dermatologic diagnoses [28]. In an East Timor deployment with 193 dermatologic diagnoses 9/27/1999–2/20/2000, there were no scabies infections [5]. However, a scabies outbreak occurred in 2015 with 340 new cases of scabies in the French military following a national increase in scabies incidence across France [62]. Prevention of scabies includes avoiding skin to skin contact or sharing items with an infected person, and prophylactic treatment upon exposure [64]. Treatment includes permethrin 5% cream or malathion. An important aspect of treatment is washing all bedding and clothes, and prophylactic treatment of close contacts. Treatment failure is usually due to inadequate permethrin cream application, re-infestation, or permethrin resistance [29]. Permethrin resistance is due to mutations in the voltage-sensitive sodium channel in neurons of scabies mites [65]. In a Korean retrospective cohort study 1/2017–12/2020 of 138 scabies infestations with 38 (27.5%) treatment resistant infections, limited mobility (OR = 3.46, 95% CI) and topical steroid use prior to scabies diagnosis (OR = 3.65, 95% CI) were associated permethrin resistant infections [66].

### Other

Lymphatic filariasis (LF) is a parasitic infection transmitted by mosquitos that is manifested acutely by fever, lymphangitis, lymphadenopathy, and scrotal inflammation and chronically by lymphedema, elephantiasis and hydrocele. LF affects approximately 70 million globally. During World War 2 (WW2) many US soldiers were infected with LF, with the highest number of cases occurring in the central pacific. In Samoa, 70% of exposed troops contracted LF, and in Tonga there were 532 cases of LF in servicemen per year [67,68]. In a 2013–2014 survey and blood-slide based study of 16,467 Indian military personnel, 70 soldiers were positive for LF, with a decreasing positivity rate of 0.65% and 0.28% between 2013 and 2014 respectively, likely due to treatment given to positive cases throughout the study [69]. Another study of 531 Chilean soldiers serving in Haiti, found that 10 tested positive for LF *via* IgG ELISA test, and concluded that the risk of contracting LF in endemic areas is low in those using appropriate preventative measures such as DEET based skin repellent, permethrin based repellent, weekly 500 mg chloroquine dose, and sleep protection [70]. We did not find any studies that reported the tropical diseases onchocerciasis, buruli ulcer and yaws in military personnel.

## Insect bites

**Figure 6.**
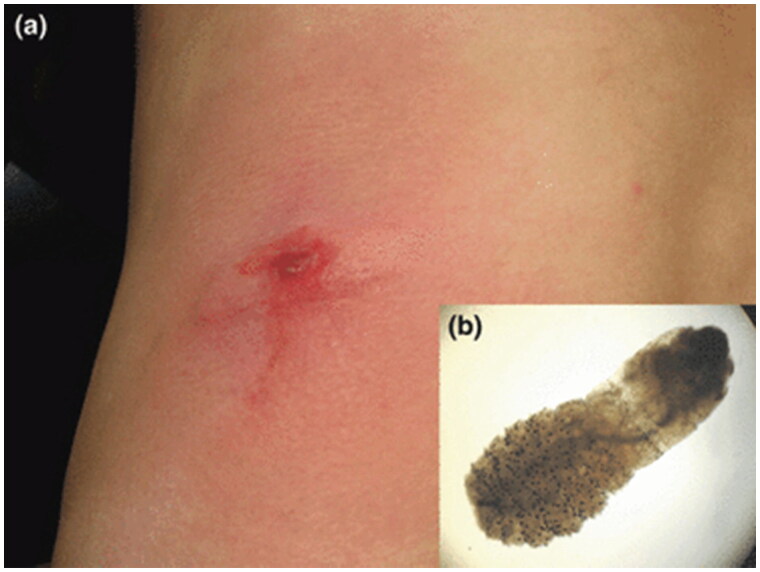
Cutaneous myiasis. Erythematous nodule with Central pustulation (a) and larva of C. anthropophaga, 7 mm long with an oval body and numerous black spines (b). Reprinted with permission from: Deng et al. [74], with permission from John Wiley and Sons.

Insect bites are common in soldiers, especially during operations and in the deployed setting (Tables 1 and 2). Multiple skin abrasions, sometimes accompanied by bullae, were detected in several French soldiers deployed to Gao, Mali, from 6/2014–10/2014. The bullae typically presented in the mornings and were due to toxigenic *Cyaneolytta* species blister beetles. The number of cases increased from 0–1 cases/month during May-July to 1–2 cases/month during August-October, the rainy season in Gao. Treatment included cleaning, debriding, and silver sulfadiazine dressings, followed by povidone iodine ointment in paraffin tulle gras dressings [71]. Cutaneous myiasis, caused by bite and larvae of the *Cordylobia anthropophaga* (Figure 6) (African Thumb Fly), was described in a British soldier serving in Sierra Leone in 2003. He was treated with antiseptic washing and one tablet of the broad spectrum antibiotic co-amoxiclav (250 mg/125 mg) every eight hours for five days to prevent abscesses [72]. In a battalion in 2007 Bangkok, 91% (*n* = 226/249) of Thai military soldiers experienced Paederus dermatitis, an ICD caused by Paederus beetles during an outbreak, with 34.1% of lesions presenting as bulla (*n* = 77/226), 33.6% as erythematous rash (*n* = 76/226), 20.8% as pustules (*n* = 47/226) and 17.3% as a ‘kissing lesion’ (*n* = 39/226). Treatment was supportive with oral antihistamines and topical steroids [73]. Preventive measures against insect bites can include long sleeve clothing, bed nets, and the use of fluorescent lights [71,73].

## Sexually transmitted infections (STIs)

STI’s were the 6th leading cause of military dermatologic consultations in a review of skin conditions in military operations [9]. Of 2696 dermatologic diagnoses made in a dermatology clinic in Iraq 1/15/2008–7/15/2008, 5% (*n* = 136) were STI’s. Of the 136 STI diagnoses, condyloma accuminata that failed to resolve with initial treatment with topical imiquimod or 0.5 podofilox was the leading cause for a dermatological consultation. The soldiers were subsequently treated with liquid nitrogen and 25% podophyllin by dermatologists, with excellent response rate (2.3 visit mean clearance) [28]. In a 2013–2021 report of the US Armed Forces, the most common STI’s in active service members were (in decreasing order of frequency) chlamydia, human papilloma virus (HPV), gonorrhea, genital herpes simplex virus (HSV), and syphilis [75].

Chlamydia was the most common STI in the US in 2020, with a rate of 481.3 cases per 100,000 population [76]. 2013–2021 chlamydia incidence rates among US Armed Forces service members were 197.6 per 10,000 p-yrs. Female vs. male service members had three times the annual incidence rates of chlamydia. 2013–2019, rates of chlamydia among both female and male US Armed Forces service members increased, with the highest rates reported in 2019 (546.0 per 10,000 p-yrs and 188.3 per 10,000 p-yrs, respectively), mainly due to increased incidence in service members under 25 years old [75]. Gonorrhea was the second most common STI in the US in 2018, with a rate of 179.1 cases per 100,000 population [77]. Gonorrhea incidence rates among US Armed Forces service members were 31.7 per 10,000 p-yrs 2013–2021. 2013–2018, rates of gonorrhea increased, and 2018–2021 rates decreased, in all groups except Black service members. In Black service members gonorrhea rates continued to increase 2013–2020 and then slightly decreased in 2021 [75]. In 2018, syphilis rates in the US were 10.8 per 100,000 [77]. Syphilis incidence rate among US Armed Forces service members was 5.0 per 10,000 p-yrs 2013–2021. Rates increased 2013 (3.2 per 10,000 p-yrs) to 2021 (6.1 per 10,000 p-yrs) in US Armed Forces service members, mostly due to an increase in cases among male service members [75].

The National Health and Nutrition Examination Surveys (NHANES) reported a Vaccine-Preventable HPV (VP-HPV) subtypes seropositivity rate of 12.9% in the US among men ages 14–59 2003–2006 [78]. In a study on the seroprevalence and sero-incidence of STI’s among US military personnel, the sero-incidence of VP-HPV 2000–2010 was 34% for the 199 male service members [79,80]. Seroprevalence of HSV in the US population among 14–49-year-olds 1999–2004 was 57.9% and 17.2% for HSV-1 and HSV-2 respectively [81]. In a study of HSV seropositivity of 1094 military personnel ages 18–30 years old 1989–2005, sero-incidence rates at or before military enlistment were 40.7% and 7.5% for HSV-1 and HSV-2, respectively [82]. In a study investigating of HSV seropositivity rates among 200 male and 200 female US service members 2006–2010, HSV-1 and HSV-2 seropositivity in female service members was 57.5% and 16.5% respectively, and 8% were positive for both (increase from rates of 39%, 4.0% and 1.5% respectively at enlistment). In male service members, seropositivity at the end of the 4-year study was 42.5%, 6%, and 3%, respectively (an increase from rates of 33.5%, 1.5% and 1.5% respectively at enlistment) [79]. HSV infection rates are not higher in young adult military personnel than civilians, nevertheless, HSV and other STIs are a major cause of morbidity in military soldiers [82].

The high prevalence of HPV and HSV in military soldiers highlights the importance of preventative measures in this population [79]. HPV vaccination benefits both the solider and the healthcare system in decreasing burden and costs [80]. However, HPV vaccination rates in active military members are low. Only 26.6% of eligible active servicewomen and 5.8% of servicemen initiated the HPV vaccination series in 2007–2017. Of those who initiated HPV vaccination, only 46.6% servicewomen and 35.1% completed the series [83]. While the military offers coverage through military insurance, HPV vaccination is not mandatory for US military service [84]. While there are several clinical trials for vaccines against genital herpes, currently there is no available HSV vaccine [85], and also no vaccines for chlamydia, gonorrhea, and syphilis [86]. Therefore, condom use with every sexual encounter decreases the risk of contracting STIs [87]. Yet, in a 2014 DoD Health Related Behaviors Survey, 36.7% of active service members reported having sex without a condom with a new partner in the last year [83]. The US Navy and Marine Corps promotes sexual responsibility and condom access, stocks condoms in minimum medical allowance lists, and provides free condoms in some settings [88].

**Figure 7.**
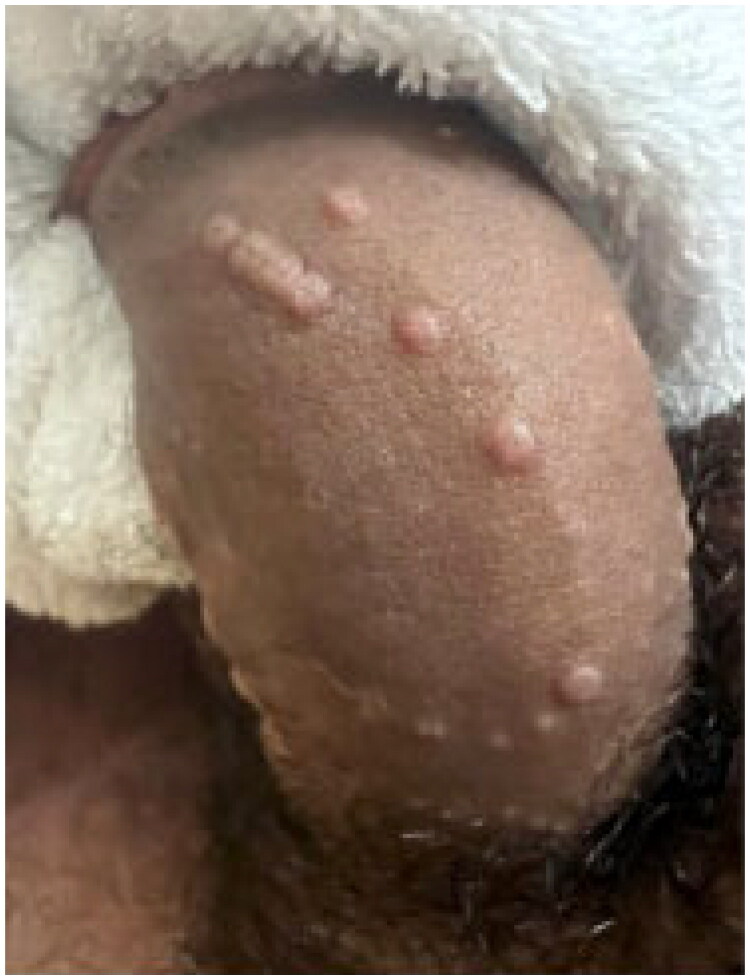
Monkeypox. Umbilicated papules on the penile shaft. Reprinted with permission from: Vallée et al. [96], with permission from Elsevier.

A new outbreak of monkeypox, an orthopoxvirus of the same family as smallpox, occurred in 5/2022. Close physical contact is a means of transmission, and in the most recent outbreak, was associated almost exclusively with sexual contact [89,90]. Monkeypox exanthems most commonly appear on the face, extremities, and groin and first presents as maculopapular eruptions that turn into vesicles that later crust (Figure 7) [91]. Monkeypox cases have been described in military soldiers [92]. Five unlinked cases of monkeypox in soldiers occurred during a monkeypox outbreak in British service personnel in 2022. [93]. By 8/2022, 40 monkeypox cases were diagnosed in the US military. The Navy and Marine Corps Public Health Center has a system in place to track monkeypox cases across all military branches and determined that monkeypox risk in the military is low [91]. There is no current approved treatment for monkeypox, but the antiviral medications tecovirimat, brincidofovir, and cidofovir used to treat smallpox are sometimes used. Intravenous vaccinia immune globulin, used to treat smallpox vaccine complications may also be authorized for use as treatment for monkeypox [94]. The smallpox vaccine confers protection from monkeypox and smallpox infections and is recommended by the CDC for certain vulnerable population [95].

## Skin cancer

### Skin cancer

There is increased prevalence of skin and keratinocyte cancers, compared to the general population, in military veterans who have served in active duty (Tables 1 and 2) [97–100]. In the general population melanoma accounts for 1% of all skin cancers, and in an observational study of skin biopsies of the Walter Reed National Military Center, melanoma cases accounted for 5% (*n* = 54) of the 1084 cases of skin malignancy 8/2015–7/2016 [99,101]. Malignant neoplasms were the second most common post-evacuation diagnosis in 170 military personnel removed from deployment in Central and Southwest Asia 1/2003–12/2006 (*n* = 13) [6]. During OIF, a US army operated dermatology clinic in Iraq reported that 8% of the 2696 total visits (*n* = 205) 1/15/2008–7/15/2008 were for skin cancer, including basal cell carcinoma (*n* = 70), squamous cell carcinoma (*n* = 68), mycosis fungoides (*n* = 1), and melanoma (*n* = 9), while 14% of visits (*n* = 357) were for benign skin tumors [28,102]. In an on-site dermatology clinic in Iraq, all benign lesions and most of the malignant lesions in Iraq were promptly treated [102].

In their retrospective cohort study of 1545 White active duty military personnel diagnosed with melanoma 1990–2004, there was a lower risk of melanoma in White military personnel 20–44 years old (Incidence RR < 1.0), and increased risk in those older than 45 years old (Incidence RR > 1.0) compared to the general population (both *p* < .05) [103]. A 2001–2015 surveillance study of 2233 melanoma diagnoses in the US military showed an exponential increase in malignant melanoma diagnoses relative to active service years, with a 12.8% increase with each additional year from 1st to 20th service years (*R*^2^ = 0.97) [104]. The increased risk of melanoma is also military branch related, with the highest risk in US AF personnel [103–108]. Airline pilots and cabin crew have approximately twice the risk of melanoma and other skin cancers as compared to the general population in a 2019 meta-analysis (of mostly studies from the 1970s and 1990s [109]. Airline pilots are exposed to high average annual effective doses of radiation (3.07), and while modern airplane windows filter most ultraviolet (UV) B radiation, more than half of UVA radiation is unfiltered [110]. A review of skin cancer in the US military published in 2021 emphasized that increased UV radiation exposure, inadequate utilization of sun-protection, and lack of education on risks for UV exposure were the leading factors contributing to the risk of skin cancer in military personnel [97].

### Sunburn

Sun exposure in military soldiers is often substantial due to spending long periods of time outdoors, which may also increase incidence and degree of photodermatitis [111]. Prevention strategies include use of sunscreen with sun protective factor 30 or higher and hats, clothing that covers large areas of skin, and constructing shade [112]. Two survey-based studies, one of 212 OIF and Operation New Dawn (OND) veterans 12/2013–5/2014, and the other of 356 AF personnel 3/2013–5/2013, reported that the majority (64% in both studies) of military personnel spent most of their days working in direct sun exposure, with only 13% and 11% respectively reporting regular use of sunscreen [100,105,113]. Furthermore, in the 2013–2014 study of OIF and OND veterans, only 30% of participants reported having regular access to sunscreen [113]. A 2016 US Army regulation for preventing environmental causalities states that it is the responsibility of brigade commanders to ensure soldiers have an adequate supply of sunscreen and that they use it daily [114].

In a 2008–2013 surveillance study of the US AF that there were lower sunburn rates in the deployed vs. non-deployed settings, which may be influenced by less time for recreational activities and more frequent wearing of military uniforms [115]. In a survey study of 212 veterans of OIF and OND 12/2013–5/2014, 63% had at least one sunburn during deployment which increased to 74% in those spending >6 h of the workday in the sun. Furthermore, 43% had 2+ sunburns, and 20% experienced blistering sunburns [113]. In a 2002–2013 surveillance study of non-deployed US military personnel, 19,172 cases (crude incidence rate of 124.8 per 100,000) of sunburn were diagnosed, with higher incidence in females (female to male RR = 1.4), White (White to Black RR = 11.7), and younger service members (≤19 y.o to 45+ age groups RR = 10.4) [112].

## Acne

Acne is a common skin disease whose pathogenesis includes follicular hyperproliferation, excess sebum production, inflammation, and *Propionibacterium acnes* bacterial growth [116]. Acne is especially common in the adolescent and young adult population, affecting 85% of 12–24 years olds. Since a large percentage (40.7% in 2018) of active-duty US military members are 25 years old or younger, acne is commonplace in the military (Tables 1 and 2) [116–118]. Acne was diagnosed in 35.7% (*n* = 470) of 1321 male Korean soldiers ages of 19–24 4/2010–9/2010 and in 10.36% (*n* = 23) of 222 Norwegian soldiers (with no specific age group noted) 9/1996-5/1997 presenting with skin disease to the Oslo Military Clinic [11,23]. In a cross-sectional study of common skin diseases in the Korean military 4/2010–9/2010, acne prevalence was associated with increased time spent in the military (*p* = .022) [23].

Acne and acne treatment can be especially challenging for military soldiers who are often required to maintain military grooming standards, are exposed to mechanical irritation, have reduced opportunity for personal hygiene, and have specific work conditions which may conflict with treatment [119]. Many military soldiers returning from Iraq experienced moderate to severe cystic acne especially prominent in areas with chronic skin irritation from heavy military gear. In addition to the mechanical irritation experienced by wearing heavy garments, the hot weather conditions in Iraq and the decreased opportunity for hygiene likely contributed to acne development [120]. Soldiers may have restricted treatment options due to potential side effects that may interfere with military duties. For example, military members in specialized military roles, such as pilots and submariners, may be disqualified from military duties when receiving treatment with isotretinoin, often making them undeployable [117,121]. Minocycline, which may cause central nervous system side effects, is considered a non-waiverable medication in the AF and thus is prohibited for treatment of aircrew service members [122]. Doxycycline may not be advisable in environments with high UV indices due to potential photosensitivity [121]. Preventative measures include skin hygiene, regular shampooing, avoidance of face touching, and reduced sun exposure, which may be difficult to maintain in a military settings [123].

## Military grooming practices- effects on the skin

### Acne keloidalis nuchae (AKN)

To maintain uniformity in the military the US Army has grooming policies that emphasize neatly groomed hair. In male service members, hair must be tapered in appearance, meaning the hair must conform to the outline of the head [124]. Likely associated with military grooming policies, diseases of hair and hair follicles were the second most prevalent skin condition in deployed US soldiers 2008–2015 accounting for 7.9%–15.16% of 429,837 dermatologic diagnoses [10]. Male military hair grooming practices may contribute to the development or exacerbation of AKN [119,125]. AKN is more common in black males, with a prevalence of 0.5–13.6% in African-Americans and a male to female ratio of 20:1. Frequent haircuts may contribute to the pathogenesis of AKN [119,126]. Prevention of AKN progression may be difficult in military settings and includes avoiding mechanical irritation from clothes, and use of antimicrobial cleansers. Treatment involves a combination of steroids (topical, intralesional or systemic) and retinoids or antibiotics [125].

### Alopecia

In female service members with long hair, defined as extending below the collar, hair must be neatly fastened or pinned above the collar [124]. Female military hair grooming practices may contribute to traction alopecia in female soldiers. Traction alopecia is associated with tight hairstyling. It is frequently seen in black and Hispanic women but can affect all races [127]. Avoiding tight hair styles is imperative for preventing permanent hair loss [128]. Military grooming practices may also exacerbate alopecia areata (AA). In a 2015 retrospective study of 1148 military conscripts and 2234 civilians, AA was significantly more common in the military population (5.4% vs. 1.7% *p* < .001) than civilians. In another retrospective study of 1658 dermatological consult of Chinese peacekeepers in Lebanon between 2007-2014, AA was one of the top five complaints and accounted for 5% of diagnoses [129]. However, in a 2020 DoD effort to support inclusivity and diversity, the US Air Force and US Army authorized female servicemembers to wear longer braids and ponytails [130].

### Pseudofolliculitis Barbae (PFB)

**Figure 8.**
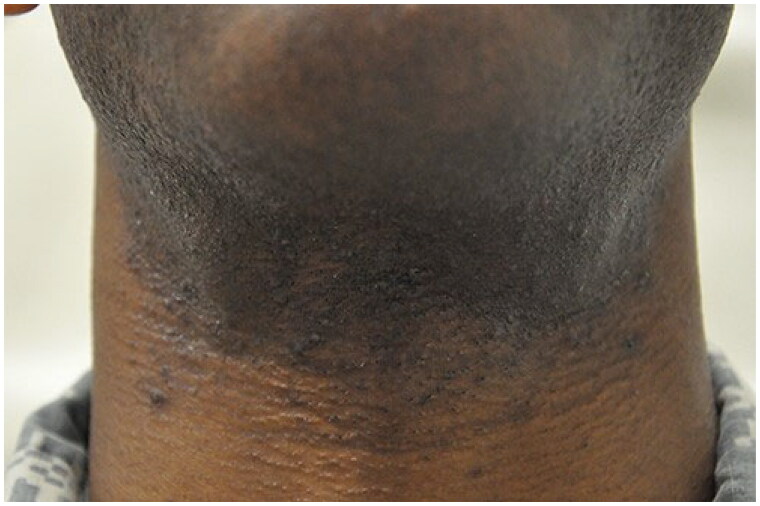
Pseudofolliculitis Barbae. Follicular papules and hyperpigmented patches. Reprinted from public domain United States Government document Tshudy and Cho [131].

As a general requirement, the US Army grooming policies emphasize a clean-shaven face when in uniform or on duty. Medical treatment may permit beard growth, though maximum authorized length must be specified [124]. PFB is associated with facial hair shaving and qualifies for receipt of a military ‘shaving waiver’ (Figure 8) [131,132]. PFB predominantly affects 45% of African American service members [10,131,133,134], which might be due their higher prevalence of helical or spiral shaped hair [132]. PFB rates decreased from 5.9% of dermatologic consults in the Vietnam War to 1.8% in OIF and less than 1% in more recent deployments, likely due to new treatment guidelines, shaving wavers, and military PFB clinics [9,131]. Treatments for PFB include grooming techniques, topical treatments (such as tretinoin and benzoyl peroxide/clindamycin combination), shaving waivers, and laser therapy [131]. However, topical treatments can cause irritation, and permanent shaving wavers are often not permitted in the military (temporary shaving wavers are allowed). Laser therapy is associated with higher rates of complications in darker skinned individuals, including postinflammatory hyperpigmentation and scarring [131]. Prevention is achieved by avoiding the trigger—shaving, however, for military soldiers who need to maintain a clean-shaven face, proper shaving techniques should be emphasized. Electric hair clippers are less likely to cause PFB. Other techniques include maintaining at least 1 mm of hair left behind after shaving, pre- and post- shaving care, and avoiding skin stretching or dry shaving [123].

## Managing skin conditions in soldiers

The US military follows strict medical eligibility criteria for enlisting soldiers, and disqualifies those with certain medical conditions, including chronic skin diseases, to protect the individual health of the applicant and ensure mission accomplishment in combat settings [135]. However, soldiers with mild cases may receive a waiver [136]. Chronic skin conditions can be especially difficult in the deployed setting. In a study of a United Nations peacekeeping mission in Darfur 3/2014–2/2015, 47.6% of the 542 soldiers with dermatologic disease were diagnosed before deployment [18]. In their paper on military evacuations due to skin conditions 1/1/2003–12/31/2006, McGraw & Norton advised that pre-deployment military screening be performed for those with a history of chronic skin conditions, such that soldiers can be counseled on preventative measures and flares, or be recommended to avoid deployment [6].

Diagnosis of atopic dermatitis (AD) after the 12th birthday is a disqualifying medical criterion for enlistment in the US military, though mild and well controlled cases often receive a waiver [135,137]. AD often flares in soldiers [6], and correlates with stress levels experienced in the military. In a cross-sectional survey-based study of 1321 Korean soldiers 4/2010–9/2010, stress levels (measured with a 14-part questionnaire) was associated with both AD and seborrheic dermatitis (SD) (*p* = .003 in both) [23]. Furthermore, environmental factors and lack of access to certain care measures, such as regular baths and moisturizers can exacerbate AD in soldiers [136]. AD can causes ocular complications and poses a risk for secondary infections, which can be especially problematic in military settings [138]. Treatment options of AD in soldiers include emollients, cleansers, and topical steroids and immunomodulators, and biologics. UV therapy can be used in non-deployed settings. Immunosuppressant use in soldiers is limited to short-term treatment due to their side effect profiles, need for laboratory monitoring, and immunosuppression [137].

A diagnosis of psoriasis renders an applicant ineligible for military admission, however psoriasis can present after a soldier has already enlisted [135]. In a study of dermatological diagnoses of US soldiers in the deployed setting 2008–2015, 0.8% of 429,837 deployed medical dermatological diagnoses and 4.0% of 452 teledermatology diagnoses were psoriasis [10]. Psoriasis can cause discomfort and koebnerization when wearing body armor and make it difficult to wear military boots and helmets. Topical steroids, and steroid sparing agents may be used in military soldiers to treat psoriasis [137]. Apremilast, is another treatment option, with no adverse effects on deployability [137,139]. Treatment with a biologic restricts a military soldier from deployment, but can be used in the non-deployed setting [137].

## Dermatitis

**Figure 9.**
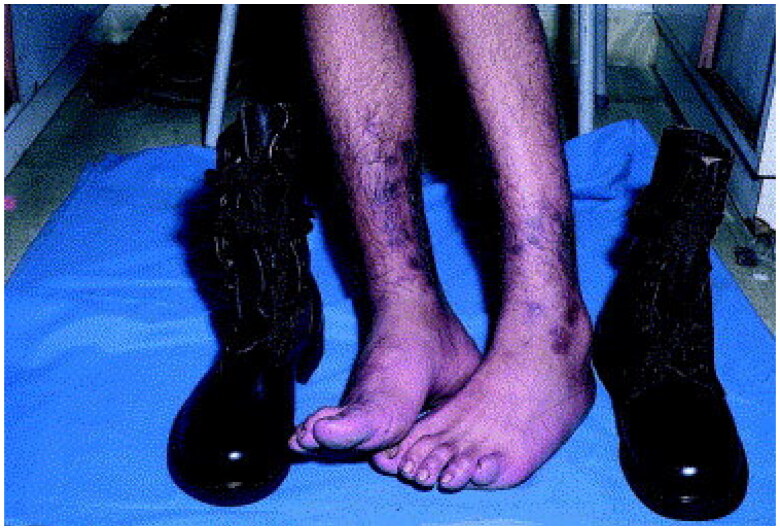
Contact dermatitis of the leg and dorsal foot secondary to military boots. Erythematous plaques correspond to the outline of the military boots. Reprinted with permission from: Oumeish and Parish [40], with permission from Elsevier.

Dermatitis and eczematous conditions are common amongst military personnel who are exposed to weather extremes, psychological stress, and irritants and allergens (Tables 1 and 2) [9,18]. In a study of 170 US soldiers evacuated from military theater due to an unknown dermatologic condition 1/1/2003–12/31/2006, 20% (most common) received a dermatitis diagnosis post-evacuation [6]. CD and other eczematous conditions accounted for 7.7% and 0.8%, respectively, of the 429,837 dermatologic diagnoses in deployed US soldiers 2008–2015 (Figure 9) [10]. In studies of military missions to Lebanon (1/2018-5/2019), Iraq (1/2008–7/2008), and Sudan (3/2014–2/2015), dermatitis and eczema were the most common skin diagnoses accounting for 27.1% (*n* = 149/549), 17% (*n* = 462/2696), and 38.7% (*n* = 210/542) of cutaneous diagnoses, respectively [17,18,28]. In contrast, in the tropical and humid environment of East Timor, 16% (*n* = 31/193) of dermatological conditions diagnosed 9/27/1999–2/20/2000 were dermatitis, mostly CD [5].

In a 2007–2009 retrospective cohort study of 18,749 military conscripts in Singapore, CD affected in 0.4 conscripts/year and mostly affected the lower limbs and hands [12]. In another retrospective study 1989–1999 of 77 occupational dermatoses in the Singapore army, ICD was 4.4 times more common than Allergic CD (ACD) [140]. ICD in military soldiers is most frequently due to alcohols, oils, grease, disinfectants, soaps and cleaners, petroleum, solvents, and wet work [140–143]. ACD occurs in military soldiers most commonly from exposure to military uniforms, camouflage, industrial fluids, shrapnel, and standard military vaccinations [141]. Military soldiers with ACD had a significantly higher atopy rate and a shorter duration of dermatitis compared to civilians in a 2018 Israeli study of textile and shoe ACD [144]. Untreated CD limits a soldier’s ability to participate in certain military duties and may lead discharge from the military. Therefore, proper diagnosis and management of both ICD and ACD is crucial [141]. Management involves avoidance of the causative agent, topical steroids, antihistamines, topical immunomodulators (such as tacrolimus and pimecrolimus), and systemic corticosteroids for severe cases [145–147]. The foundation of managing CD is prevention by educating the soldier about agents that may trigger CD [148].

## Onychocryptosis

Ingrown toenails, also known as ungus incarnatus and onychocryptosis, occur due to the nail plate piercing the nail fold causing an inflammatory reaction [149]. Risk factors include repetitive toe trauma, nail injury, constricting footwear, and lack of grooming [150]. In a 2014 retrospective prevalence study of 1148 male soldiers and 2234 male civilians evaluated at a dermatology clinic in Merzifon Military Hospital in Turkey, onychocryptosis prevalence was higher in the military group (3.3% VS. 0.3% *p* < .001) [151]. Treatment of mild or moderate cases may be treated with open-toe footwear, managing underlying hyperhidrosis and onychomycosis if present, warm water soaks, gutter splints, cotton nail casts or metal braces. Recalcitrant and severe cases may be treated with phenol matricectomy, and is also more effective at preventing recurrence than non-surgical interventions [150,152].

## Extreme weather conditions

Extreme weather conditions may affect soldiers who spend a larger portion of their day unsheltered in extreme cold or heat. Weather related injuries can have long-lasting effects on soldiers involving disfigurement and disability [153]. However, most weather related skin injuries may be avoided with education, proper use of protective clothes and footwear [111,154].

**Figure 10.**
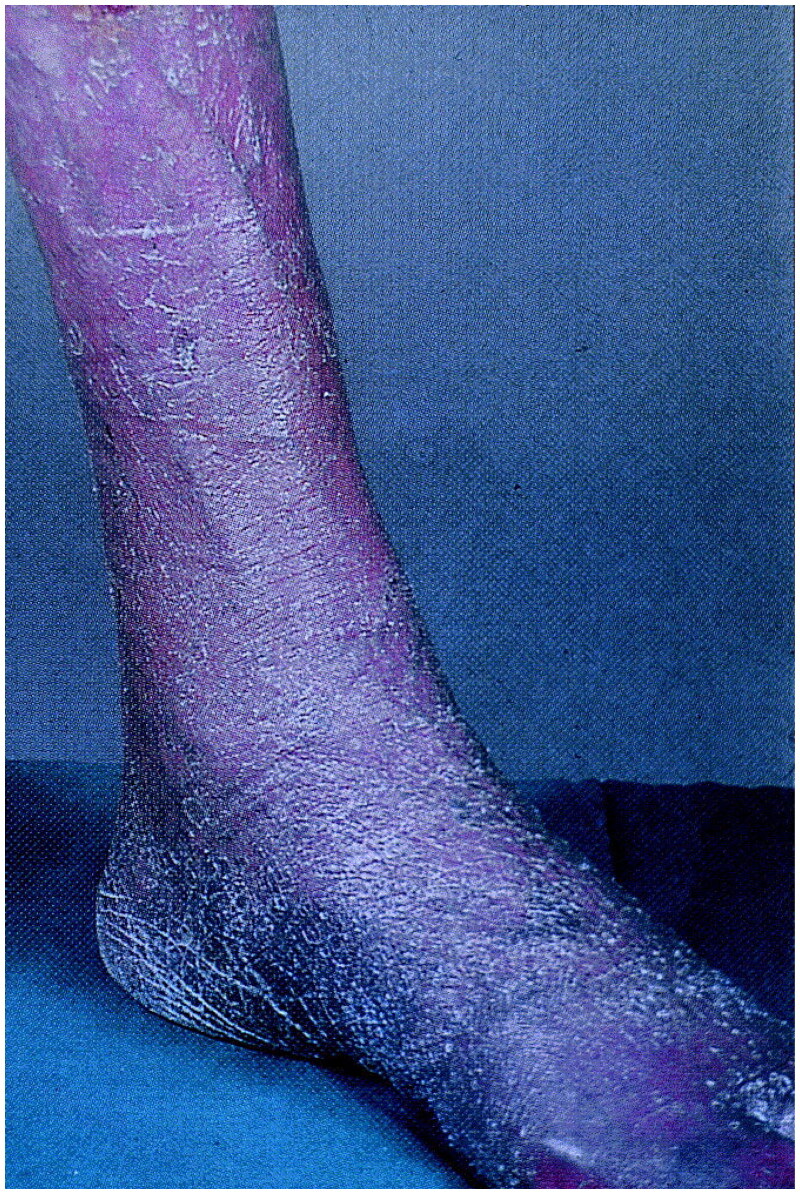
Frostbite of the lower leg and foot. Reprinted with permission from: Oumeish and Parish [40], with permission from Elsevier.

In a US military surveillance study of 2524 cold injury cases 2009–2014, overall cold injury incidence rates were 36.2 per 100,000 p-yrs, with frostbite being the most common type, accounting for 1226 cold injury cases in all 4 military units (Figure 10). Rates were higher in female service members with the most striking difference between incidence rates of male and female service members in the Army (85.4 vs 53.6 per 100,000 p-yrs, respectively). Cold injury incidence rates were higher among Black vs. White service members, especially in the Army (rates = 106.5 vs. 46.0 per 100,000 p-yrs, respectively) and Marine Corps (rate = 93.2 vs. 35.6 per 100,000 p-yrs, respectively) [155]. Frostbite is a common cold injury in military soldiers and most commonly affects the digits, ears, nose, and facial skin. Often, the severity of frostbite injury is not evident until a day or two after warming the tissue [153]. Other common cold weather injuries in military soldiers include immersion foot, pernio, Raynaud phenomenon, and cold urticaria [156]. Weather injuries due to UV irradiation and heat can cause acute sunburn and might exacerbate preexisting skin conditions or result in photoreactions from medications [111].

## Occupational exposure

Occupational and warfare exposures may cause skin diseases in soldiers. For example, in the Iran-Iraq conflict, sulfur mustard (SM) was used for chemical warfare, with an Iranian military study of 40 soldiers exposed to SM 1983–1988, reporting hyperpigmentation (55%), xerosis (40%), cherry angiomas (37.5%), atrophy (27.5%), and hypopigmentation (25%) [157]. There is evidence that organochlorine exposure during the Vietnam war is correlated with chloracne [158]. ICD from direct skin contact to JP-5 jet fuel was reported in a 19-year-old US navy male sailor that was treated betamethasone valerate 0.1% ointment twice daily for two weeks followed by emollients three times daily for 4 weeks [159]. Overall, 73,725 cases of plant dermatitis were reported in an 11-year surveillance study of US soldiers 1/1/2010–12/31/2020, with a crude rate of 5.3 per 1000 p-yrs, with higher rates among male service members (5.7 per 1000 p-yrs) and those in combat-related occupations (11.9 per 1000 p-yrs). White service members had an incidence rate of 7.5 per 1000 p-yrs compared 1.1 per 1000 p-yrs in Black service members. Case numbers were higher May to September (67% of all cases) and in Georgia (*n* = 12,874), California (*n* = 8764), North Carolina (*n* = 7707) and Virginia (*n* = 7125) [160]. Prevention of occupational exposures in soldiers, who are vulnerable to different hazards than the general population, should include monitoring programs that use biomarkers to evaluate exposure [161].

## Future directions

Implementation of telemedicine can aid in diagnosing skin disease in military soldiers [162,163]. In a 2004–2012 retrospective study of the Department of Defense (DoD) teledermatology consultation program, teledermatology accounted for 40% of 10,817 teleconsultations, of which 84% were in military soldiers. Teledermatology requests were consulted in an average time of 5 h and 14 min, and prevented 46 evacuations [164]. Use of mobile clinics as facilities for procedures may be a solution for managing dermatoses in soldiers, and were effective during the COVID-19 pandemic [165].

## Study limitations

This review is subject to certain limitations. As the focus of this review is on military populations, we were limited to including only military health information shared publicly. As most of the literature we found described the experiences of the US military, this review disproportionally includes studies focused on US soldiers. While several of the studies included in this review are from other countries, more studies exploring dermatological diseases affecting military populations throughout the world are needed. Studies on prevalence of lichen planus, hidradenitis suppurativa, and rosacea in military populations are outdated and limited.

## Conclusions

Skin disease is amongst the most frequent reasons for soldiers and military personnel to seek medical care and can have a significant impact on soldiers’ wellbeing and ability to fulfill military duties. Military settings affect the presentation and spectrum of cutaneous disease in soldiers and the potential treatment and prevention options. Dermatitis and eczematous conditions were the most prevalent conditions in areas with hot and humid climates. Certain triggers of skin disease related to military standards, stress, and work-related exposures may be unavoidable in the military population. Some treatments used in the general population (such as isotretinoin, minocycline, biologic agents, immunosuppressant agents) may restrict military soldiers from fulfilling their military duties. Implications for military settings should be considered before initiation of treatment.

## Data Availability

Data sharing is not applicable to this article as no new data were created or analyzed in this study.
